# Artificial Intelligence Applications for Assessment, Monitoring, and Management of Parkinson Disease Symptoms: Protocol for a Systematic Review

**DOI:** 10.2196/46581

**Published:** 2023-06-14

**Authors:** Katie Bounsall, Madison Milne-Ives, Andrew Hall, Camille Carroll, Edward Meinert

**Affiliations:** 1 Peninsula Medical School University of Plymouth Plymouth United Kingdom; 2 Centre for Health Technology University of Plymouth Plymouth United Kingdom; 3 University Hospitals Plymouth National Health Service Trust Plymouth United Kingdom; 4 Translational and Clinical Research Institute Newcastle upon Tyne United Kingdom; 5 Department of Primary Care and Public Health Imperial College London London United Kingdom; 6 Harvard T.H. Chan School of Public Health Harvard University Boston, MA United States

**Keywords:** artificial intelligence, machine learning, Parkinson disease, Parkinson, neurodegenerative, review method, systematic review

## Abstract

**Background:**

Parkinson disease (PD) is the second most prevalent neurodegenerative disease, with around 10 million people with PD worldwide. Current assessments of PD symptoms are conducted by questionnaires and clinician assessments and have many limitations, including unreliable reporting of symptoms, little autonomy for patients over their disease management, and standard clinical review intervals regardless of disease status or clinical need. To address these limitations, digital technologies including wearable sensors, smartphone apps, and artificial intelligence (AI) methods have been implemented for this population. Many reviews have explored the use of AI in the diagnosis of PD and management of specific symptoms; however, there is limited research on the application of AI to the monitoring and management of the range of PD symptoms. A comprehensive review of the application of AI methods is necessary to address the gap of high-quality reviews and highlight the developments of the use of AI within PD care.

**Objective:**

The purpose of this protocol is to guide a systematic review to identify and summarize the current applications of AI applied to the assessment, monitoring, and management of PD symptoms.

**Methods:**

This review protocol was structured using the PRISMA-P (Preferred Reporting Items for Systematic Reviews and Meta-Analyses Protocols) and the Population, Intervention, Comparator, Outcome, and Study (PICOS) frameworks. The following 5 databases will be systematically searched: PubMed, IEEE Xplore, Institute for Scientific Information’s Web of Science, Scopus, and the Cochrane Library. Title and abstract screening, full-text review, and data extraction will be conducted by 2 independent reviewers. Data will be extracted into a predetermined form, and any disagreements in screening or extraction will be discussed. Risk of bias will be assessed using the Cochrane Collaboration Risk of Bias 2 tool for randomized trials and the Mixed Methods Appraisal Tool for nonrandomized trials.

**Results:**

As of April 2023, this systematic review has not yet been started. It is expected to begin in May 2023, with the aim to complete by September 2023.

**Conclusions:**

The systematic review subsequently conducted as a product of this protocol will provide an overview of the AI methods being used for the assessment, monitoring, and management of PD symptoms. This will identify areas for further research in which AI methods can be applied to the assessment or management of PD symptoms and could support the future implementation of AI-based tools for the effective management of PD.

**International Registered Report Identifier (IRRID):**

PRR1-10.2196/46581

## Introduction

Parkinson disease (PD) is the second most prevalent neurodegenerative disease worldwide, with around 10 million people with PD worldwide [1]. This number represents a 2.5-fold increase in prevalence over the past generation [2,3]. PD is a neurological disorder associated with motor and nonmotor features [4] affecting multiple aspects of movement, including planning, initiation, and execution [5]. Management of PD is very complex over the course of the disease due to its progressive nature [6]. Developments of digital technologies have allowed for the remote, objective monitoring of symptoms, allowing care teams to take data-driven approaches to care, producing large amounts of data [7]. Artificial intelligence (AI) methods have been applied to data sets to improve the timeliness of care for patients, assist with analysis, and reduce health care provider burden [8]. The existing research has primarily focused on the application of AI methods in the diagnosis of PD or the management of motor symptoms [9,10], despite nonmotor symptoms (NMS) causing a significant burden for people with PD, as well as reduced quality of life, and being the main contributor to the overall cost of care [11,12]. It is important to understand how the range of AI methods can be applied to all symptoms experienced by people with PD to aid with monitoring and disease management, as well as assisting the patient to understand their disease profile more clearly and facilitate better self-management [13].

PD is most commonly characterized by a resting tremor, bradykinesia, and rigidity, accompanied with changes in posture and gait. During the development of PD, motor symptoms are observed prior to cognitive and behavioral changes, such as dementia [14]**.** Despite being defined as a movement disorder, people with PD can also be affected by a wide range of symptoms spanning beyond the classic motor symptoms [4]. NMS affect all people with PD and can include constipation, urinary dysfunction, sleep disturbances, and depression [15,16]. The National Institute for Health and Care Excellence acknowledges that NMS are considered a significant burden for people with PD, and their recognition and management is an important yet unmet need of PD [17]. Evidence shows that NMS can occur at any stage of the disease, with some NMS occurring prior to the onset of motor symptoms [18]. Generally, the assessment and characterization of both motor and NMS rely on clinical assessment with follow-ups occurring every 6-12 months for stable patients, according to the National Institute for Health and Care Excellence guidelines [19]. The appropriate assessment of symptoms affecting people with PD is vital for an effective clinical management and managing symptom severity [20,21]. This assessment methodology aims to allow for both the subjective and objective assessment of the impact of PD to achieve a clear overall impact of the disease for people with PD [22]; however, this is not always achieved. These assessments lack granularity during disease or medication cycles; these assessments represent only a small portion of a patient’s life and may not adequately capture the nuances of a highly variable condition [7,23]. The quality of the information collected is limited by patients’ and care partners’ recall about their recent symptom burden, which can be worsened by the cognitive impairments associated with PD. An international survey published in 2010 highlighted that 62% of patients with PD do not declare NMS including pain, sexual difficulty, sleep dysfunction, or bowel incontinence either due to embarrassment or not being aware that such symptoms are linked to PD [16]. As a result, some symptoms, particularly NMS, are often overlooked or undeclared by people with PD, which results in incorrect disease management, impacting upon quality of life for both the patient and their care partner, and increased care needs and costs [16].

The remote monitoring of PD symptoms has become increasingly common because it can allow patients to self-manage their condition at home [24-26]. Digital technologies allow for the at-home monitoring of patients through smart devices, sensors, and other tools. At-home monitoring allows for the continuous oversight of disease presentation in an objective manner to ensure no symptoms are forgotten, overlooked, or misinterpreted [23,27]. Several studies have proposed and evaluated the use of wearable devices and smartphones with built-in sensors for monitoring both motor and NMS for people with PD [27-30]. By enabling long-term data collection in a nonclinical setting, digital technologies can reduce the burden on clinical data collection and allow clinicians to spend contact time with patients more meaningfully. The wealth of available data and improved clinical efficiency has the potential to facilitate the diagnosis, monitoring, and management of PD. The present applications of wearable devices include assessing gait (including freezing of gait), tremor, bradykinesia, rigidity, sleep dysfunction or disturbance, blood pressure, and detecting falls [31]. Data from accelerometers and gyroscopes on various parts of the body can also allow for the estimation of the Unified Parkinson’s Disease Rating Scale scores and determine bradykinesia in both scripted and unscripted conditions [32-35]. The analysis of such large amounts of data, however, can be extremely time-consuming and subject to variation in analyses [23].

The implementation of AI is being extensively used in a variety of health care and medical contexts, including biomedicine, diagnostics, living assistance, biomedical information process, and biomedical research [36-38]. In PD, AI has been explored for both diagnostic support and disease monitoring. Wearables combined with machine learning algorithms have been reported to sufficiently discriminate between healthy participants and patients with PD, with an accuracy of over 90%. The prediction of symptom severity using motor symptoms and various algorithms and sensors has also reported high accuracy results [39]. Specific data sets that have been used to support clinical decision-making in PD care include handwriting patterns [40], gait [41], and neuroimaging [42]. At present, the application of AI appears to be primarily focused on the motor aspects of the disease, despite some NMS data being routinely recorded via wearable or smart devices [43]. The assessments of NMS in PD carry the same limitations as any other semiobjective rating scale, including intra- and interobserver inconsistencies and bias. Further, many NMS are not as easy to track remotely in the way motor symptoms are; for example, multiple motor symptoms, including tremor, bradykinesia, and gait or freezing of gait are detectable using gyroscopes and accelerometers usually embedded within wearable devices. Due to the large amount and wide range of NMS, as well as accessibility for the assessment of the symptom, a large portion cannot be objectively identified in the same way. Multiple research studies have focused on NMS being present in early disease states and are therefore focused on use for diagnostic purposes [44-46]. The tracking of NMS using digital technologies reduces the likelihood of the patient or carer misreporting symptoms and provides a much larger data set, eradicating the issue with clinical assessments providing only a *snapshot* in time. Implementing AI methods for NMS assessment and management can contribute toward improving patient and carer quality of life by addressing NMS more appropriately [47], staging people with PD [48], providing more tailored care [49], and potentially reducing the likelihood of NMS being overlooked.

A vast number of publications and systematic reviews have assessed the application of AI into the diagnosis of PD [9,39]. Additional reviews have focused on the management of individual symptoms [50], assessment using wearables [31], individual treatment options in relation to machine learning [51], or both diagnosis and management [21,39]; however, no systematic reviews were identified that comprehensively identify the use of all AI methods specifically for disease and symptom management following diagnosis. A search of PROSPERO and PubMed for a range of keyword combinations including “artificial intelligence,” “machine learning,” “deep learning,” and “Parkinson” identified a range of different reviews; however, none focused on AI for PD symptom and disease management.

To address this lack of comprehensive reviews, this review aims to identify the current uses of all AI applications across the assessment, management, and monitoring of symptoms of PD and identify the challenges and pitfalls of the current application of AI in PD. To achieve this aim, we will provide a comprehensive overview of the current state of the literature; assess and compare the type of AI applied; and identify the challenges and barriers and provide recommendations for the use of AI within this population.

## Methods

### Overview

The methodology for this review has been generated in accordance with the PRISMA-P (Preferred Reporting Items for Systematic review and Meta-Analysis Protocols) [52], as displayed in Multimedia Appendix 1, as well as the population, intervention, comparison, outcomes, and study (PICOS) framework [53,54].

### Eligibility Selection Criteria

The PICOS-type framework, as detailed in Table 1, is based on the research aim stated above.

**Table 1:** Population, intervention, comparison, outcomes, and study (PICOS) framework.

Population	People diagnosed with PD^a^
Intervention	AI^b^ tools, including all machine learning approaches and deep learning approaches for the assessment, management, or monitoring of PD symptoms.
Comparator	No comparator is required.
Outcomes	The primary objective is to identify and summarize the AI methods for the assessment, monitoring, and management of PD symptoms. Therefore, the primary outcome will be to identify and compare the different AI methods used in the assessment, monitoring, and management of PD symptoms. Comparison between models will be made using accuracy data. The secondary outcome is to identify the current challenges, barriers, or pitfalls with the application of AI in PD.
Study types	Qualitative, quantitative, and mixed methods studies that use any form of AI in the assessment, monitoring, or management of PD symptoms will be eligible for inclusion. Reviews, protocols, and papers that describe interventions without evaluating them will be excluded.

^a^PD: Parkinson disease.

^b^AI: artificial intelligence.

### Search Strategy

Five databases will be searched for appropriate publications—PubMed, IEEE Xplore, Institute for Scientific Information’s Web of Science, the Cochrane Library, and Scopus. The databases were chosen as they were frequently searched in previous systematic reviews on PD and machine learning and have a wide range of coverage of digital technology and health. The following search terms were developed following an initial review of the literature and used to develop the search terms and search strategy. Search items will include Medical Subject Headings (MeSH) terms and keywords related to machine learning, PD, and symptom management. The search terms that will be used in this review are grouped into 3 themes (Table 2). The search string will be created using the following structure: “Artificial intelligence” (MeSH or keyword) AND “Parkinson’s disease” (MeSH or keyword) AND “intended use” (MeSH or keyword). Multimedia Appendix 2 shows a sample search string. The reviews will be hand searched for relevant studies.

**Table 2:** Search terms.

Category	MeSH^a^	Keywords (in title or abstract)
Artificial intelligence	“Artificial intelligence” and “Machine learning”	“Artificial intelligence” OR “machine learning” OR “deep learning” OR “neural network” OR “neural networks” OR “supervised machine learning” OR “unsupervised machine learning” OR “reinforcement learning”
Parkinson disease	“Parkinson’s disease”	“Parkinson’s disease” OR “Parkinson’s” OR “Parkinson” OR “Parkinson disease”
Intended use	N/A^b^	“assessment” OR “monitoring” OR “management”

^a^MeSH: Medical Subject Headings

^b^N/A: not applicable.

### Inclusion Criteria

Any study will be included that uses any AI method (ie, machine learning, deep learning, or algorithm) to detect (ie, the identification of symptoms), monitor (ie, maintain an objective oversight of symptoms), or manage (ie, use of symptoms data to reduce symptom burden, reduce prevalence of symptom, or support clinical decision-making) any symptom of PD. The AI method will not be limited and will include all machine learning, deep learning, or algorithm approaches. No restrictions will be placed on the symptom in question; however, a diagnosis of PD must be present. Studies with any type of sampled population will be eligible for inclusion, with no restrictions on age, gender, or country. Interventions with comparisons to control groups with no intervention, irrelevant interventions, or minimal interventions will all be included.

### Exclusion Criteria

Due to the progressive nature of the digital health field with continuous adaptations being made, in addition to a clear increase in the number of papers published from this date, any study published before 2010 will be excluded [55]. Studies that address the use of AI in the diagnosis of PD or the management of symptoms related to PD without a diagnosis of PD (eg, Alzheimer disease, sleep disturbance, etc) will be excluded. Studies that assess symptoms in the context of diagnosis or assess related symptoms with a different neurological diagnosis will be excluded. Studies written in any other language aside from English will be excluded, as the research team do not have the capabilities to read them or the resources to have them translated. Studies that focus on Parkinsonian conditions (eg, progressive supranuclear palsy and multiple system atrophy) that are not PD will be excluded. Reviews, protocols, and papers that describe interventions without evaluating them will be excluded (Textbox 1).

**Inclusion and exclusion criteria.**

**Inclusion criteria**

- Year: 2010 to present
- Article type
  - Detection, monitoring, or management of one or more symptoms of Parkinson disease (PD) using any artificial intelligence (AI) method
  - Randomized controlled trials; qualitative, quantitative, cohort, and case studies
- Language: English
- Population
  - Human studies of any gender, age, or country
  - Diagnosis of PD

**Exclusion criteria**

- Year: any article published before 2010
- Article type:
  - Studies that assess symptoms in the context of diagnosis
  - Symptoms of PD without a diagnosis
  - Reviews, protocols, practice guidelines, commentaries, letters, editorials, conference abstracts, and posters without full texts
  - Studies that assess related symptoms with a different neurological diagnosis
  - Studies using Parkinsonian conditions that are not PD (eg, multiple system atrophy or progressive supranuclear palsy)
- Language: non-English
- Population: animal models

### Screening and Article Selection

The references returned from the database searches will be exported into the citation management software EndNote 20 (Clarivate) for storage and duplicate removal. The screening will take place in the following three stages: (1) EndNote’s search function will be used to screen the articles using keywords in comparison to the inclusion and exclusion criteria, and exclude any studies that are evidently ineligible; (2) the remaining references (titles and abstracts only) will be screened by 2 independent reviewers; and (3) the full texts of the studies will then be screened by 2 independent reviewers to determine the final set of papers for inclusion. Any disagreement between the reviewers will be discussed until a consensus is achieved. If a consensus cannot be achieved, a third reviewer will be consulted to determine the outcome. Details of the screening and selection process will be recorded in a PRISMA-P [52] flow diagram to ensure reproducibility of the study. Stage 1 EndNote 20 searches will be recorded and included in the final review as an appendix.

### Data Extraction

The full text of each article included in the final set will be assessed by 2 independent reviewers to extract the data outlined in Table 3. As outlined above, any discrepancies between reviewers will be discussed and resolved by an additional reviewer if a consensus cannot be achieved.

**Table 3:** Article information and data extraction.

Article information	Data to be extracted
General study information	Year of publication Country of study Sample demographics (including age, gender, and target population) Initial sample size Analyzed sample size
Model characteristics	AI^a^ method Delivery of the AI method (eg, app, web portal, etc) Aim of intervention Target user or audience of intervention (eg, people with PD^b^, PD care partners, or HCPs^c^) Purpose of the AI method (ie, symptom assessment, monitoring, and management) Symptoms assessed Data collection method or technique (eg, wearable sensor, smartphone app, etc)
Evaluation or validation	Effectiveness, accuracy, specificity, or sensitivity of method (if reported) Test training sets of data and their percentages Effectiveness, efficacy, usability, acceptability, and feasibility outcome measures (if reported)

^a^AI: artificial intelligence.

^b^PD: Parkinson disease.

^c^HCP: health care provider.

### Quality Appraisal and Risk of Bias Assessment

Risk of bias for all of the included studies will be independently assessed by 2 reviewers. Any discrepancies between reviewers will be discussed and resolved by a third reviewer if necessary. For randomized controlled trials, risk of bias will be assessed using the Cochrane Collaboration Risk of Bias 2 tool [56,57]. For nonrandomized studies, the Mixed Methods Appraisal Tool will be used [58] due to its appraisal of most common study types and designs.

### Data Analysis and Synthesis

If there are sufficient studies, we plan to conduct a meta-analysis of outcome measures; however, there is a possibility that it may not be feasible due to the expected heterogeneity of studies. If meta-analysis is possible, the design will be guided by [59], and heterogeneity will be assessed using *I*^2^ statistic [60]. The primary outcome for meta-analysis will be classification accuracy of symptoms and is expressed at a proportion between 0 and 1 (eg, percentage rate and area under the curve). The extracted data will be summarized using descriptive analysis to provide the outcomes measured, machine learning tools used, and symptoms assessed. The risk of bias in the studies will be considered in the synthesis.

### Ethical Considerations

There are no ethical issues of concern in this secondary analysis of published data.

## Results

As of April 2023, database searches have not yet begun. The full systematic review is expected to be completed and submitted for publication by September 2023.

## Discussion

### Principal Findings

This paper describes the protocol for a systematic review to identify the current uses of AI for people with PD in the assessment, monitoring, and management of PD symptoms. The results from this review will improve knowledge about the way PD is managed using AI methods. With increasing need for the effective management of the wide-ranging symptoms of PD and the accelerated use of computational technologies in PD care, a comprehensive analysis of the research surrounding the use of these tools for symptom assessment, monitoring, and management is necessary. This review will identify how AI is currently being used with this population, as well as its potential benefits and limitations.

There has been a recent increase in the focus of digital health and computational technologies for the diagnosis and management of PD, and several reviews have been conducted [10,31,39,61]. This review aims to be the first to summarize the evidence of the use of AI for disease and symptom management for PD. This review differs from the aforementioned reviews as it places no restriction on the symptom in question; for example, in [61], the authors focus on freezing of gait or on the diagnosis of PD only. The results from this review will therefore provide a more thorough overview of the management of PD using AI methods and the potential benefits to the patient, health care professional, and service.

### Limitations

A limitation from this review that may impact the conclusions we are able to make is the inclusion of all PD symptoms and their assessment or management via AI. As there are reportedly over 40 symptoms of PD [62], some symptoms may feature more heavily within the literature than others; therefore, conclusions for certain symptoms may be easier to draw than others. It is anticipated that there will be a high level of heterogeneity of outcomes in addition to the inclusion of qualitative and quantitative studies, which may result in difficulty when comparing the effects. When extracting data, it may be possible to compare the effects in subgroups (eg, symptom type, gender, etc). Finally only studies written in the English language will be included in this review; therefore, relevant articles may be missed as a result.

### Conclusions

From this review, we anticipate that the findings will provide insight into the current uses of AI in disease management for people with PD and may inform future research or developments for the implementation of AI-based tools for PD management from research to practice. As this review has the potential to impact upon technological advancements and clinical management, the results will be disseminated through peer-reviewed publication.

## Appendix

Multimedia Appendix 1 PRISMA-P (Preferred Reporting Items for Systematic review and Meta-Analysis Protocols) 2015 checklist.

Multimedia Appendix 2 Sample search strings.

## Abbreviations

AI: artificial intelligence
MeSH: Medical Subject Headings
NMS: nonmotor symptoms
PD: Parkinson disease
PICOS: Population, Intervention, Comparator, Outcome, and Study
PRISMA-P: Preferred Reporting Items for Systematic Reviews and Meta-Analyses Protocols

## Data Availability

Data sharing is not applicable to this article as no data sets were generated or analyzed during this study. Data sets generated and analyzed from the prospective full review will be available from the corresponding author upon request.
