# Supplementary material for: Artificial Intelligence Applications for Assessment, Monitoring, and Management of Parkinson Disease Symptoms: Protocol for a Systematic Review
Source: JMIR Res Protoc. 2023 Jun 14;12:e46581. doi: 10.2196/46581 (PMC10337354; doi:10.2196/46581)
Supplement: Multimedia Appendix 2 [file resprot_v12i1e46581_app2.docx]

## Sample search strings.

| Database | Search string | References |
| --- | --- | --- |
| PubMed | ((((("Artificial intelligence"[MeSH Terms] OR "machine learning"[MeSH Terms] OR ("Artificial intelligence"[Title/Absratc] OR " ai " [Title/Abstract] OR "machine learning"[All Fields] OR "deep learning"[Title/Abstract] OR "algorithms"[Title/Abstract] OR "neural network"[Title/Abstract] OR "neural networks"[Title/Abstract] OR "supervised machine learning"[Title/Abstract] OR "unsupervised machine learning"[Title/Abstract] OR "reinforcement learning"[Title/Abstract])) AND "Parkinson disease"[MeSH Terms]) OR ("Parkinson's disease"[Title/Abstract] OR "Parkinson's"[Title/Abstract] OR "Parkinson"[Title/Abstract] OR "Parkinson disease"[Title/Abstract])) AND ("assessment"[Title/Abstract] OR "monitoring"[Title/Abstract] OR "management"[Title/Abstract])) NOT (diagnosis[Title/Abstract])) NOT (rat[Title/Abstract] OR rodent[Title/Abstract] OR mouse[Title/Abstract] OR mice[Title/Abstract] OR horse[Title/Abstract] OR pig[Title/Abstract] OR dog[Title/Abstract]) | 8523 |
| IEEE Xplore | ("Mesh_Terms":artificial intelligence) OR ("Mesh_Terms":machine learning) OR ("Abstract":"artificial intelligence" OR "Abstract":" ai " OR "Abstract":"machine learning" OR "Abstract":"deep learning" OR "Abstract":"algorithms" OR "Abstract":"neural network" OR "Abstract":"neural networks" OR "Abstract":"supervised machine learning" OR "Abstract":"unsupervised machine learning") AND ("Mesh_Terms":Parkinson disease) OR ("Abstract":"parkinson's disease" OR "Abstract":"parkinson's" OR "Abstract":"parkinson" OR "Abstract":"Parkinson disease") AND ("Abstract":"assessment" OR "Abstract":"monitoring" OR "Abstract":"management") NOT ("Abstract":diagnosis) NOT ("Abstract":rat OR "Abstract":rodent OR "Abstract":mouse OR "Abstract":mice OR "Abstract":horse OR "Abstract":pig OR "Abstract":dog) | 4476 |
| Scopus | ( TITLE-ABS-KEY ( "artificial intelligence" OR "machine learning" OR "deep learning" OR "algorithms" OR "neural network" OR "neural networks" OR "supervised machine learning" OR "unsupervised machine learning" OR "reinforcement learning" ) AND TITLE-ABS-KEY ( "Parkinson's disease" OR "Parkinson's" OR "Parkinson" OR "Parkinson disease" ) AND TITLE-ABS-KEY ( "assessment" OR "monitoring" OR "management" ) AND NOT TITLE-ABS-KEY ( diagnosis OR rat OR mouse OR rodent OR mice OR horse OR pig OR dog ) ) AND PUBYEAR > 2009 AND PUBYEAR > 2009 | 1358 |
| Web of Science | “artificial intelligence" OR "machine learning" OR "deep learning" OR "algorithms" OR "supervised machine learning" OR "unsupervised machine learning" OR "reinforcement learning" (Topic) and "Parkinson's disease" OR "Parkinson's" OR "parkinson" or "parkinson disease" (Topic) and "assessment" OR "monitoring" OR "management" (Topic) not diagnosis OR rat OR mouse OR rodent OR mice OR horse OR pig OR dog (Topic)” | 447 |
| Cochrane Library | "artificial intelligence" OR "machine learning" OR “deep learning” OR "algorithms" or "neural network" OR "neural networks" OR "supervised machine learning" OR "unsupervised machine learning" OR "reinforcement learning" in Title Abstract Keyword AND “Parkinson’s disease” OR "Parkinson’s" OR "Parkinson" OR “Parkinson disease” in Title Abstract Keyword AND "assessment" OR "monitoring" OR "management" in Title Abstract Keyword NOT diagnosis OR rat OR mouse OR rodent OR mice OR horse OR pig OR dog in Title Abstract Keyword - with Cochrane Library publication date Between Jan 2010 and Mar 2023 (Word variations have been searched) |  |
